# Evolution of Cardiac Damage Across Clinically Defined Stages of Aortic Stenosis in Patients Undergoing TAVR: A Single-Center Retrospective Cohort Study

**DOI:** 10.3390/jcm15041575

**Published:** 2026-02-17

**Authors:** María Rivadeneira-Ruiz, Carmen Olmos, Sandra Gil-Abizanda, Pilar Jiménez-Quevedo, Eduardo Pozo-Osinalde, Luis Nombela-Franco, José Alberto de Agustín, Fabián Islas

**Affiliations:** 1 Departamento de Cardiología, Hospital Clínico San Carlos, Instituto de Investigación Sanitaria del Hospital, Clínico San Carlos (IdISSC), 28040 Madrid, Spain; 2 Facultad de Medicina, Salud y Deportes, Universidad Europea de Madrid, C/Tajo, 28670 Madrid, Spain; 3 Servicio de Cardiología, Hospital Universitario de La Zarzuela, C/de las Pléyades, 25, 28023 Madrid, Spain

**Keywords:** aortic stenosis, cardiac damage, echocardiography, global longitudinal strain, transcatheter aortic valve replacement, risk stratification, observational cohort

## Abstract

**Background:** Echocardiography is essential for diagnosing and guiding therapy in aortic stenosis (AS). Cardiac damage staging systems may better characterize myocardial and extracardiac involvement. We aim to evaluate the presence and progression of cardiac damage across the clinical course of AS. **Methods:** A single-center retrospective cohort study was conducted, which included consecutive patients who ultimately underwent TAVR and had retrievable serial echocardiograms at moderate AS, first severe AS, and pre-TAVR symptomatic severe AS (2017–2021). A total of 179 patients were evaluated (mean age 82.7 [5.9] years at moderate AS; 46% male, *p* = 0.27). Cardiac damage was classified according to two established staging systems. **Results:** The median time from moderate to severe AS was 32 months (IQR 18–48). Most echocardiographic parameters deteriorated primarily at symptom onset, whereas moderate AS and first severe (asymptomatic) AS showed broadly similar profiles. However, left ventricular global longitudinal strain (LV GLS) was already impaired at the first severe stage, and right ventricular–arterial coupling (RVAc, TAPSE/sPAP) progressively worsened as AS advanced to the severe stage, independently of symptom status (LV GLS −18.1%, n = 163; −17.1%, n = 143; and −14.9%, n = 143; RVAc 1.0, n = 131; 0.8, n = 130; and 0.7, n = 130), respectively; overall *p* < 0.05. Both staging systems demonstrated increasing cardiac damage with AS progression. **Conclusions:** Cardiac damage may occur early in AS. The marked deterioration at symptom onset underscores the importance of systematic myocardial assessment and supports prospective studies to evaluate whether integrating LV GLS and RVAc as sensitive early markers of disease progression improves risk stratification.

## 1. Introduction

Degenerative aortic stenosis (AS) is the most prevalent valvular heart disease in developed countries and its burden will continue to rise as populations age. Meta-analytic estimates in adults ≥ 75 years report a prevalence of any AS of 12.4% and severe AS of 3.4%, underscoring a substantial and growing public-health challenge [1]. Notably, contemporary population data across the spectrum of untreated AS demonstrate a high mortality risk even at non-severe stages, supporting the need for earlier diagnosis, closer surveillance, and evaluation of timely intervention strategies [2,3].

Contemporary guidelines emphasize echocardiography as the cornerstone for grading severity and guiding management, with intervention recommended in symptomatic severe AS—either at baseline or after symptoms elicited during exercise testing [4,5]—or in asymptomatic patients with LV ejection fraction (LVEF) < 50% without an alternative explanation. Nonetheless, a sizeable fraction of patients remains asymptomatic despite advanced disease, and undertreatment persists in community cohorts, even among severe AS [6,7,8]. Observational studies indicate that up to 40% of individuals with severe AS are asymptomatic at diagnosis [9].

To refine risk stratification beyond conventional gradients and valve area, staging systems of cardiac damage have been proposed. The widely recognized framework by Généreux et al. characterizes extravalvular involvement—from LV damage to left atrial/mitral, pulmonary vasculature/tricuspid, and right ventricular damage—and associates higher stages with worse outcomes after valve replacement [10]. More recently, simplified models have incorporated earlier markers of myocardial injury such as global longitudinal strain (GLS) and right ventricular–arterial coupling (RVAc), which may improve prognostic stratification in patients referred for transcatheter aortic valve replacement (TAVR) [11].

Parallel developments have focused on asymptomatic severe AS and on moderate AS. Randomized trials in carefully selected asymptomatic severe AS populations suggest that earlier intervention can reduce composite events or hospitalizations, whereas other trials did not demonstrate a clear mortality benefit—highlighting nuances across patient selection, endpoints, and statistical power [12,13,14]. In moderate AS, emerging evidence indicates higher long-term mortality and lifetime loss compared with no/mild AS, particularly when concomitant cardiac damage is present, although population studies advise caution in generalizing risk among asymptomatic outpatients under standard surveillance [15,16,17,18].

In this context, a descriptive characterization of when subclinical myocardial and extracardiac damage emerges across clinical stages of AS—using reproducible echocardiographic markers such as GLS and RVAc—may help phenotype disease trajectories.

## 2. Objectives

The objective of this study is to describe the evolution of myocardial and extracardiac damage across three clinically defined stages of AS (moderate diagnosis, first severe diagnosis, pre-TAVR symptomatic severe), using echocardiographic parameters (especially LVEF, GLS and RVAc defined as TAPSE/sPAP), and to compare staging distributions between two cardiac damage systems [10,11], in patients with AS referred for valvular intervention.

## 3. Materials and Methods

### 3.1. Study Design and Setting

We conducted a single-center, retrospective observational study at Hospital Clínico San Carlos (Madrid, Spain). The study included consecutive patients who underwent TAVR between January 2017 and January 2021. Clinical and echocardiographic data were collected from institutional electronic health records and imaging archives. Relevant clinical and epidemiological information was retrieved from a comprehensive evaluation that was systematically carried out prior to TAVR implantation. Echocardiograms were performed either at our hospital or at referring hospitals; in the latter case, the studies were imported and reanalysed by experienced echocardiographers. This study’s protocol was approved by the Institutional Ethics Committee at our hospital, and all patients provided written informed consent. STROBE recommendations for observational studies were followed. A complete checklist can be found as Supplementary Material (Table S1) [19].

### 3.2. Study Population

A total of 524 patients treated with TAVR within the established timeframe was initially screened. Inclusion criteria were as follows: (i) age ≥18 years; (ii) diagnosis of moderate or severe AS confirmed by comprehensive transthoracic echocardiography (TTE); and (iii) availability of retrievable TTEs at three predefined clinically relevant time points—(1) the earliest available TTE meeting criteria for moderate AS, (2) the first TTE meeting criteria for severe AS while classified as asymptomatic, and (3) the pre-TAVR TTE in the symptomatic severe AS phase.

Bicuspid aortic valve morphology was included if patients met AS criteria. Concomitant valvular disease, including moderate or severe mitral and tricuspid regurgitation, was not used as an exclusion criterion, as these conditions represent markers of extracardiac damage within the myocardial damage staging framework. Their inclusion was intentional to preserve the clinical relevance and external validity of the analysis.

**Figure 1:**
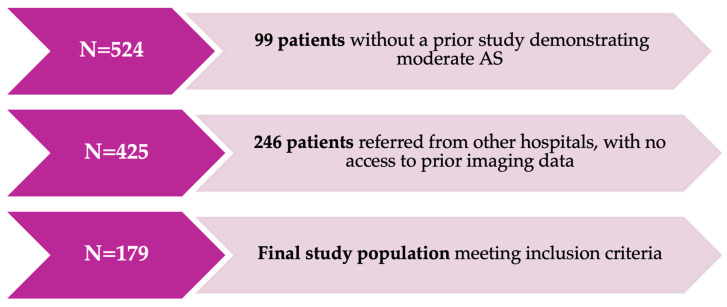
Study flow diagram.

After applying these criteria, 99 patients were excluded because their first available echocardiogram showed severe AS, with no prior study showing moderate AS. Additionally, 246 patients referred from other hospitals were excluded because they lacked access to prior imaging data. Therefore, 179 patients were included in the final analysis. (Figure 1).

### 3.3. Definitions and Classification

AS severity was classified according to established and robust echocardiographic criteria, including peak aortic jet velocity, mean transvalvular pressure gradient, and aortic valve area (AVA) calculated by the continuity equation. Left ventricular outflow tract (LVOT) diameter was measured on a zoomed parasternal long-axis view at mid-systole, inner-edge to inner-edge, parallel and adjacent to the aortic valve (at the same level used for LVOT PW Doppler), in accordance with ASE/EACVI recommendations. LVOT PW Doppler sample volume was positioned just on the LV side of the valve and adjusted to obtain a laminar flow curve [20] (Supplementary Material, Table S2).

For this study, three severity stages were defined: moderate AS, severe asymptomatic AS, and severe symptomatic AS (the latter meeting the current criteria for intervention). The pre-TAVR echocardiogram was obtained as part of routine clinical evaluation prior to intervention. This assessment reflects a clinical stage in which patients were already symptomatic and referred for valve intervention, rather than the precise onset of symptoms. Therefore, this time point represents the pre-intervention symptomatic phase and not the moment of symptom development.

Symptom status was determined retrospectively from clinical documentation (cardiology clinic notes and functional class/NYHA when available) within a pre-specified window of ±30 days around each echocardiographic examination. Exercise testing was not routinely performed to confirm asymptomatic status and was performed at the clinical’s discretion.

Two myocardial damage staging systems—those proposed by Généreux [10] and Gutiérrez [11]—were applied at each of the three stages. These classifications incorporate parameters of left-sided cardiac damage (subclinical involvement, significant systolic or diastolic dysfunction, increased LA volume, or significant mitral regurgitation) and right-sided damage (elevated pulmonary pressures, significant tricuspid regurgitation, or right ventricular systolic dysfunction). Généreux’s system comprises five stages, whereas Gutiérrez’s system comprises four. These cardiac damage staging systems were assigned at each echocardiographic time point using a pre-specified, criteria-based algorithm implemented in Stata. Stage assignment was computed automatically from extracted echocardiographic variables according to the published staging definitions and did not involve manual adjudication. Because staging was programmatic and rule-based, assessor blinding was not applicable; the algorithm did not use the chronological time point as an input beyond the measured variables required to define each stage.

### 3.4. Echocardiographic Assessment

All transthoracic echocardiograms were performed in accordance with guideline recommendations [20] using commercially available ultrasound systems (GE and Philips ultrasound systems). Measurements included LVEF, LV GLS, left atrial volume index (LAVI), transmitral Doppler parameters, pulmonary artery systolic pressure (sPAP), right ventricular function (e.g., tricuspid annular plane systolic excursion (TAPSE) or right ventricular–arterial coupling (RVAc)), and transvalvular flow rate. Studies were conducted or supervised by cardiologists specialized in cardiovascular imaging.

GLS was analyzed offline using vendor-independent software (TomTec Imaging Systems, Munich, Germany) based on 2D speckle-tracking echocardiography, following current recommendations for deformation imaging [21]. GLS was derived from standard apical views (four-, two-, and three-chamber) using the 17-segment LV model and averaged across available views. Endocardial tracking quality was assessed by visual inspection; the region of interest was adjusted when needed, and segments with clearly inadequate tracking were excluded. GLS was reported only when tracking was considered adequate in at least two apical views and ≥15/17 segments were acceptable; otherwise, GLS was treated as missing for that time point. GLS values were analyzed as signed values (more negative indicates better systolic function). Results were interpreted against contemporary reference limits (lower limit of normal around −18%) while acknowledging vendor- and load-dependency [22].

RVAc was assessed using the echocardiographic TAPSE-to-sPAP ratio, calculated as TAPSE (mm) divided by systolic pulmonary artery pressure (sPAP, mmHg), and is therefore expressed in mm/mmHg. RVAc could only be computed when sPAP was estimable from the tricuspid regurgitation jet; accordingly, RVAc was treated as missing when sPAP could not be estimated.

LVEF was assessed by the biplane Simpson method and interpreted using ASE reference ranges (normal LVEF 52–72% in men and 54–74% in women) [23].

Feasibility for key echocardiographic measures (GLS, TAPSE, sPAP and RVAc) was quantified at each time and reported as available n/N (%) (Supplementary Material, Table S3).

### 3.5. Study Measures

The primary study measures were echocardiographic markers of myocardial and extracardiac involvement (including GLS, RVAc, LVEF, sPAP, chamber volumes, and valvular regurgitation severity) assessed across the three predefined AS stages. Secondary measures included shifts in cardiac damage stage distributions (Généreux and Gutiérrez systems) across time points.

### 3.6. Statistical Analysis

Categorical variables were presented as counts and percentages and compared using the chi-square test or Fisher’s exact test, as appropriate. Continuous variables were expressed as means and standard deviations or medians and interquartile ranges and were compared using Student’s *t*-test or the Wilcoxon test, as appropriate.

To account for repeated echocardiographic measurements within individuals across the three echocardiographic assessments (moderate AS, first diagnosis of severe AS, and symptomatic severe AS prior to TAVR), longitudinal changes in echocardiographic parameters were analyzed using mixed-effects models. Continuous echocardiographic variables were analyzed using linear mixed-effects models (mixed), whereas binary variables were analyzed using mixed-effects logistic regression models (melogit). In all models, echocardiographic assessment was included as a categorical fixed effect, and a participant-specific random intercept was specified to account for within-subject correlation. Estimated marginal means for continuous outcomes and predicted probabilities for binary outcomes were obtained for each assessment, along with pairwise comparisons between assessments and corresponding 95% confidence intervals, using marginal predictions and pairwise contrasts (margins/pwcompare). An adjustment for multiple comparisons was performed using Bonferroni correction. This likelihood-based approach allows inclusion of participants with incomplete follow-up by incorporating all available observations under a missing-at-random assumption (Supplementary Material, File S1).

Missingness was summarized by time point, and sensitivity complete-case analyses and repeated-measures ANOVA analysis were also performed; results were consistent with the mixed-model findings (Supplementary Material, Table S4).

To evaluate changes in myocardial damage staging according to AS severity, a mixed-effects ordinal logistic regression model (meologit; cumulative logit proportional odds) was employed. Echocardiographic assessment was included as a categorical fixed effect, and a participant-specific random intercept was specified to account for within-subject correlation. Results are reported as odds ratios (ORs) with 95% confidence intervals (CIs), representing the odds of being in a higher damage stage. Post hoc pairwise comparisons across time points were performed using marginal predictions and pairwise contrasts (margins/pwcompare), with Bonferroni adjustment where applicable. Staging at each time point was assigned only when all pre-specified components required for the staging algorithm were available (complete-case staging). If one or more staging components were missing, the stage was coded as unclassifiable (missing) for that time point.

All *p*-values were two-sided, and statistical significance was defined as *p* < 0.05. Analyses were performed using Stata version 16 (StataCorp, College Station, TX, USA).

During the preparation of this manuscript, no scientific content was generated or altered by the AI. The authors have reviewed and edited the output and take full responsibility for the content of this publication.

## 4. Results

### 4.1. Pre-TAVR Clinical Characteristics (Index Procedure)

Of the 524 patients initially screened, 179 met the inclusion criteria and were included in the analysis. In this cohort, more than half of the patients were women (54%, *p* = 0.27), with a mean age of 82.7 [5.1] years at moderate AS; 85.8 [5.6] years at asymptomatic severe AS and 86.7 [6] years at symptomatic severe AS. The most frequent comorbidities at the time of TAVR implantation were arterial hypertension (82%) and dyslipidemia (68%). Median EuroSCORE II was 4.5 (2.4–7.5) (Table 1).

Regarding disease progression, the median time from the diagnosis of moderate AS to the first diagnosis of severe AS was 32 months (IQR 18–48), and from that point to intervention—i.e., the transition to symptomatic severe AS—the median time was 4 months (IQR 2.4–12.6). The mean time from the pre-TAVR echocardiogram to the intervention was 1.2 [1.3] months.

### 4.2. Echocardiographic Results

Analysis of echocardiographic parameters (Table 2) revealed significant differences across nearly all variables.

Several parameters deteriorated at symptom onset, whereas values during moderate AS and asymptomatic severe AS were largely comparable. LV GLS progressively deteriorated across stages, from −17.9% (−18.7–17.2) in moderate AS, to −16.3% (−17.3–15.4) in asymptomatic severe AS, and −14.9 (−15.7–14.1) once symptoms had developed. LVEF also showed a significant reduction in symptomatic severe AS, though remaining within the normal range: 64.9% (63.4–66.4) in moderate AS, 63.5% (61.4–65.5) in asymptomatic severe AS, and 58.1% (56.6–59.6) in symptomatic severe AS. Importantly, the proportion of patients with LVEF <50% increased when symptoms appeared (moderate AS: 5.7%; severe asymptomatic AS 6.2% and severe symptomatic 16%; *p* value for mixed-effects logistic regression model = 0.003). LV volumes, both end-diastolic (LVEDVi) and end-systolic (LVESVi), were significantly higher in symptomatic severe AS compared with the two earlier stages.

Certain echocardiographic variables, such as left atrial volume index (LAVI) and RVAc, were already affected once AS became severe, but did not differ according to the presence of symptoms. RVAc declined from 1.0 (0.9–1.2) in moderate AS to 0.8 (0.7–0.9) in asymptomatic severe AS and 0.7 (0.5–0.8) in symptomatic severe AS.

**Figure 2:**
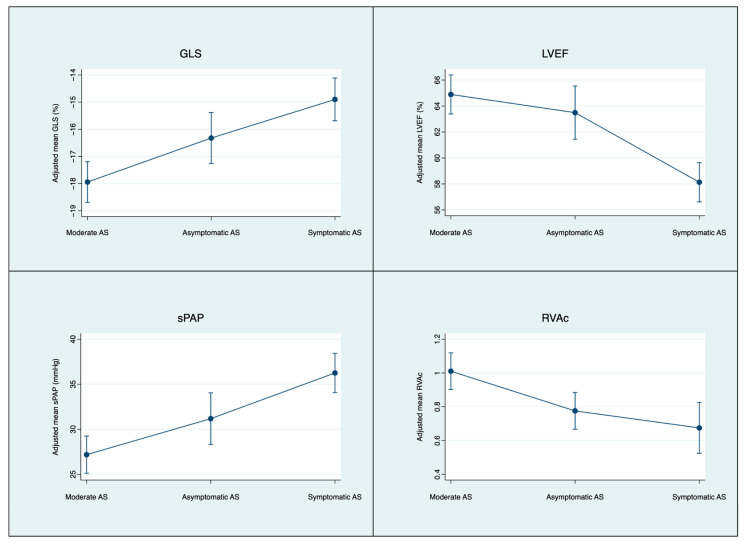
Trajectories of key echocardiographic parameters (LV GLS, LVEF, sPAP and RVAc) across the clinical course of AS. Time points correspond to the predefined echocardiographic assessment. Solid lines represent model-based estimated means derived from mixed-effects models with a patient-level random intercept to account for repeated measures; vertical bars indicate 95% confidence intervals. LV GLS is reported as signed values (more negative indicates better systolic function). RVAc was defined as TAPSE/sPAP and was treated as missing when sPAP could not be estimated. Abbreviations: AS, aortic stenosis; GLS, global longitudinal strain; LVEF, left ventricular ejection fraction; sPAP, systolic pulmonary artery pressure; RVAc, right ventricular–arterial coupling.

Likewise, pulmonary pressures progressively increased across the three stages of AS severity: 27.2 mmHg (25.1–29.2) in moderate AS, 31.2 mmHg (28.3–34.1) in asymptomatic severe AS and 36.3 mmHg (34.1–38.4) in symptomatic severe AS. (Figure 2).

Lastly, several parameters were comparable across the three stages of AS. For instance, transvalvular flow did not significantly progress within stages, diastolic filling pressures were equally elevated regardless of the severity of AS, left ventricular mass remained stable across all stages, and the proportion of patients with significant concomitant tricuspid regurgitation did not differ between groups.

These findings indicate that cardiac involvement may be present even in earlier stages of the disease, while the most significant deterioration occurs at the transition to symptomatic severe AS.

### 4.3. Myocardial Damage Staging Systems

Using the Généreux classification [10], at the time of moderate AS, 78% of patients showed left-sided cardiac damage (Stages 1–2) and 16% showed right-sided damage (Stages 3–4). In contrast, according to the Gutiérrez classification [11], 60% of patients had no significant cardiac damage (Stage 0), 35% had left-sided damage (Stages 1–2), and 5% had right-sided damage (Stage 3).

When AS progressed to severe without symptoms, Généreux staging classified 74% with left-sided damage and 24% with right-sided involvement, while Gutiérrez staging identified 67% without significant damage, 24% with left-sided damage, and 9% with right-sided damage.

**Figure 3:**
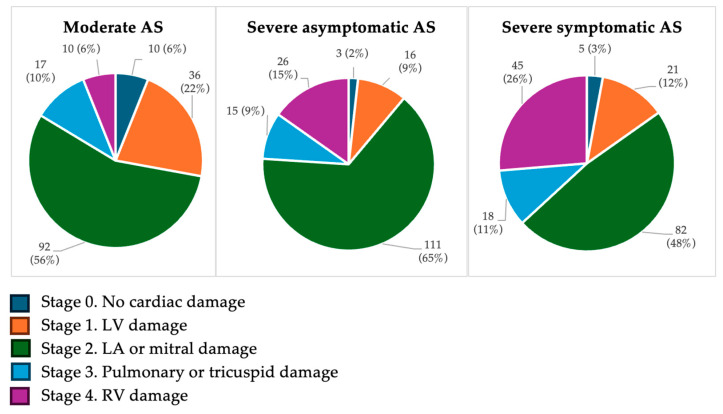
Staging classification of cardiac damage according to Généreux’s system [10]. Values are presented as number of patients (%). Percentages are calculated with each AS severity group (moderate, severe asymptomatic and severe symptomatic). Abbreviations: AS, aortic stenosis; LV, left ventricle; LA, left atrium; RV, right ventricle.

**Figure 4:**
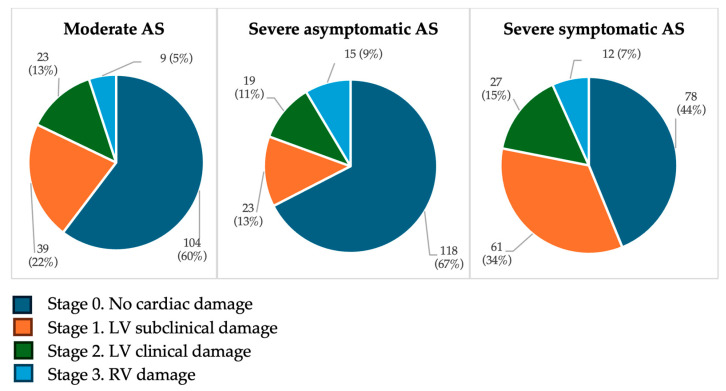
Staging classification of cardiac damage according to Gutiérrez–Ortiz’s system [11]. Values are presented as number of patients (%). Percentages are calculated with each AS severity group (moderate, severe asymptomatic and severe symptomatic). Abbreviations: AS, aortic stenosis; LA, left atrium; LV, left ventricle; RV, right ventricle.

Finally, in symptomatic severe AS, Généreux classification showed that 60% of the cohort met the criteria for left-sided damage and 37% for right-sided involvement, whereas Gutiérrez classification identified 44% without significant damage, 49% with left-sided damage, and 7% with right-sided involvement (Figure 3 and Figure 4).

Among the 179 echocardiograms available at each disease stage (moderate AS, asymptomatic severe AS, and symptomatic severe AS), the Généreux stage could be assigned in 165 (92.2%), 171 (95.5%), and 171 (95.5%), respectively (unclassifiable due to missing components: 14, 8, and 8). Gutiérrez stage could be assigned in 175 (97.8%), 175 (97.8%), and 178 (99.4%), respectively (unclassifiable: 4, 4, and 1).

**Figure 5:**
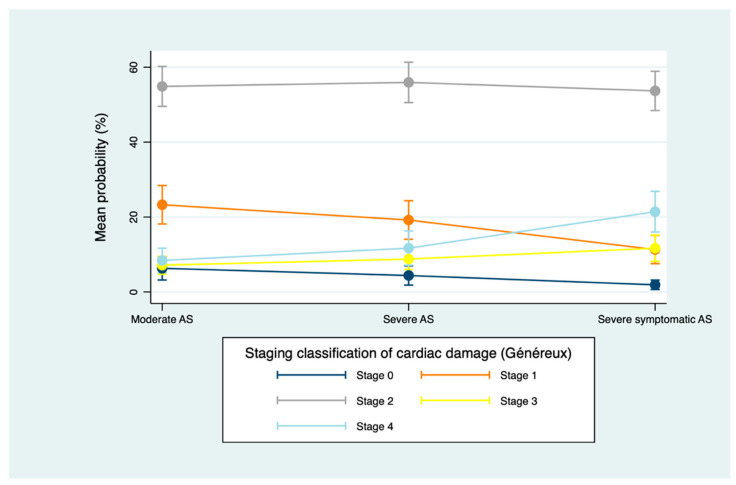
Change in cardiac damage stage by AS severity using Généreux’s system [10]. Mean predicted probabilities (unadjusted) derived from the ordinal mixed-effects model, with AS severity as the only fixed effect, accounting for within-subject correlation via a subject-specific random intercept. Abbreviations: AS, aortic stenosis. *p* value = 0.000.

**Figure 6:**
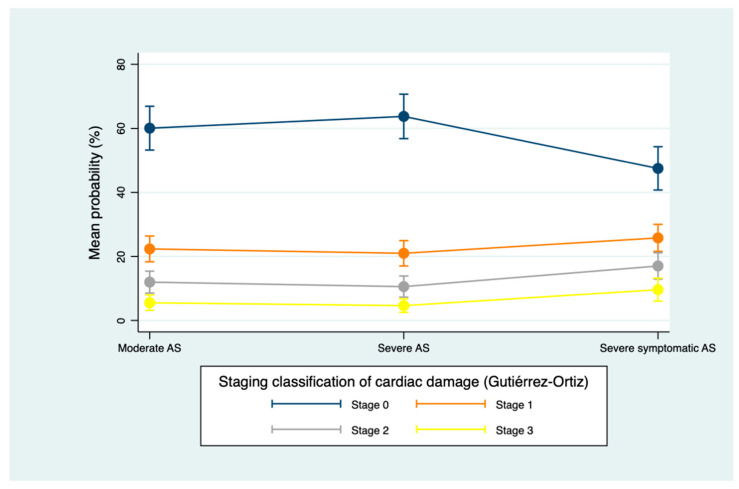
Change in cardiac damage stage by AS severity according to Gutiérrez–Ortiz’s system [11]. Mean predicted probabilities (unadjusted) derived from the ordinal mixed-effects model, with AS severity as the only fixed effect, accounting for within-subject correlation via a subject-specific random intercept. Abbreviations: AS, aortic stenosis. *p* value = 0.000.

Across both staging systems, the most noteworthy increase in cardiac damage occurred at transition to symptomatic severe AS, with no significant difference in the distribution of damage staging between moderate AS and asymptomatic severe AS (Table 3, Figure 5 and Figure 6).

## 5. Discussion

In this study, we analyzed the presence and evolution of cardiac damage at three stages in patients with AS referred for valvular intervention: moderate AS, severe asymptomatic AS, and severe symptomatic AS. The main findings of our work are as follows: (1) AS was associated with some degree of myocardial damage even at early stages; (2) symptomatic severe AS was linked to a significant deterioration in most echocardiographic parameters (LV GLS, LVEF and LV volumes), compared to the stages of moderate and asymptomatic severe AS; (3) LV GLS was already impaired in severe AS even without symptoms; (4) the majority of imaging features in moderate AS and asymptomatic severe AS were comparable; (5) Only RVAc and LAVI demonstrated significant deterioration as AS progressed to the severe stage independent of symptom status; and (6) LV GLS and RVAc may help identify early myocardial involvement in this disease.

These findings contrast with the classical view that structural cardiac impairment emerges primarily in advanced phases of the disease and are consistent with prior literature demonstrating increased mortality in some patients with moderate AS, particularly among those with specific clinical or echocardiographic characteristics [16,17].

### 5.1. Comparison of Staging Systems

In our study, the staging system proposed by Généreux [10] classifies a larger proportion of patients as having damage at earlier stages, identifying early myocardial damage in nearly all patients with moderate AS. Indeed, only 6% of our cohort exhibited no evidence of cardiac involvement at this stage. However, this may limit the system’s ability to discriminate clinically meaningful damage, diluting its prognostic utility.

Conversely, the system proposed by Gutiérrez [11] was more stringent regarding the assignment of cardiac damage. According to our data, almost 70% of patients showed no relevant cardiac damage in the early stages of AS. This tool, which incorporates more sensitive markers of left ventricular dysfunction (such as LV GLS and RVAc), enables a more refined identification of subclinical myocardial impairment.

### 5.2. Patterns of Echocardiographic Behavior

We identified several distinct patterns across the disease spectrum:

#### 5.2.1. Parameters Altered Early and Remaining Stable Throughout Disease Progression

Diastolic dysfunction and reduced transvalvular flow were present even in moderate AS. Regarding these variables, population studies have identified advanced diastolic dysfunction as a strong predictor of mortality independent of AS severity [24], whereas low transvalvular flow is one of the most robust markers of adverse outcomes in both moderate and severe AS [25]. Left ventricular hypertrophy and increased myocardial mass—particularly with concentric geometry—have also been associated with worse clinical outcomes [26].

#### 5.2.2. Parameters That Clearly Deteriorate upon Symptom Onset

These included LV volumes, LVEF, and LV GLS. In our study, we observed a significant deterioration in LV size and function parameters, which was evident only at the stage of severe AS, once symptoms had developed. Importantly, LV GLS deterioration despite preserved LVEF supports its role as an early marker of myocardial involvement in AS. In our cohort, LV GLS was preserved in moderate AS but was already impaired in severe AS even without symptoms, and remained reduced in severe symptomatic AS. These findings suggest that LV GLS may identify subclinical left myocardial dysfunction earlier than LVEF and may complement conventional parameters for risk stratification [9,11,26].

#### 5.2.3. Parameters Altered in Severe AS, Regardless of Symptoms

LAVI and RVAc fall into this category. These findings reflect chronic pressure overload and cardiac remodeling, which are characteristic even in asymptomatic patients.

Right ventricular dysfunction, as measured by conventional parameters, emerges later in the AS spectrum and are more strongly associated with poor prognosis when they occur alongside left ventricular damage [10,27,28,29].

In this case, RVAc is used as an indicator of subtle right-sided damage. This parameter has already been shown to be a better discriminator of outcomes in patients with AS than other variables related to right ventricular function [30].

### 5.3. Clinical Implications

Our results have several important clinical implications that build upon existing evidence on the progression of myocardial damage in AS.

First, moderate AS should not always be considered benign. A substantial proportion of patients in this group already exhibit structural and functional myocardial damage. These findings align with recent studies that demonstrate increased mortality in moderate AS, particularly when extracardiac damage or adverse echocardiographic parameters coexist [15,16,31,32]. Nevertheless, we should acknowledge that our cohort represents a high-risk group, as it is comprised only of patients who progressed to symptomatic severe AS.

Second, these findings support evaluating whether adding LV GLS and RVAc improves risk stratification in prospective cohorts. Advanced echocardiographic parameters—especially LV GLS and RVAc—provide incremental information beyond traditional markers of AS severity such as mean gradient, peak velocity, or aortic valve area. Their value in detecting subclinical dysfunction has been supported by recent studies that propose their integration into progression and clinical decision-making algorithms [11]. Additionally, numerous investigations have shown that these markers—particularly RVAc—are associated with increased mortality in patients undergoing TAVR, supporting their routine use not only in postprocedural assessment but also in earlier disease stages [33,34].

Furthermore, early therapeutic intervention in selected AS subgroups has been evaluated in trials such as EARLY-TAVR [14], EVOLVED [12], and AVATAR [13], which suggests that early treatment in asymptomatic severe AS may benefit selected patients. Our findings indicate that a subgroup of patients with moderate AS and early myocardial damage might also benefit from early intervention—a relevant hypothesis that warrants investigation in future prospective studies [32,35]. Stratification tools incorporating multimodal parameters—advanced imaging, fibrosis biomarkers, and calcification burden—may better address the heterogeneity of cardiac remodeling in AS and aid in determining the optimal timing of intervention [32].

Beyond cardiac damage evaluation, a comprehensive multidisciplinary assessment of patients with AS throughout the clinical process, before and after the intervention, by a multidisciplinary team targeting frailty, nutritional status and mobility is warranted to improve outcomes in this increasing population [36].

### 5.4. Study Limitations

The main limitations of our study stem from its retrospective observational design and single-center nature. Because inclusion required serial imaging available within our system, selection bias is possible. Specifically, patients referred late or without retrievable prior imaging may be under-represented, potentially enriching the cohort with slower progressors and leading to underestimation of progression magnitude/rate from moderate to severe AS. Furthermore, this study is not designed to infer causal effects of symptom onset. Symptoms may influence timing of imaging and referral.

Regarding the assessment of symptoms, exercise testing was not systematically performed, and potential symptom misclassification in patients included in the “severe asymptomatic AS” category cannot be completely ruled out.

In addition, the inclusion of patients with moderate or severe concomitant valvular disease, while enhancing real-world applicability, may have introduced heterogeneity and potential confounding in the echocardiographic assessment of aortic stenosis severity. However, these conditions were considered integral components of extracardiac damage within the myocardial damage staging framework and were therefore intentionally retained.

No assessment of intra- or interobserver variability was performed, as all the studies were conducted and interpreted by cardiologists with expertise in cardiac imaging. However, all measurements were reviewed by a single expert reader, who also carried out all LV GLS calculations. Specific image-quality-dependent parameters—particularly LV GLS—were unavailable in all examinations, and could represent a source of bias. However, no correlation with disease severity was found.

Lastly, the sample size was relatively small, limiting power for subgroup analyses.

## 6. Conclusions

Cardiac damage associated with AS may occur before it becomes severe and can be detected using simple, widely available echocardiographic parameters. In our cohort, morphological and biventricular functional deterioration became most evident at symptom onset, underlining the importance of comprehensive imaging to better characterize cardiac damage through the different stages of AS. Larger prospective studies are warranted to determine which combination of parameters best identifies patients with subclinical cardiac damage that could benefit from earlier intervention and to validate refined staging models that integrate advanced imaging and clinical data.

## Figures and Tables

**Table 1:** Clinical characteristics of the cohort at the time of the TAVR procedure.

	N = 179
**Women, n (%)**	97 (54.2)
**Body mass index, kg/m^2^**	28.4 ± 5.9
**Body surface area, m^2^**	1.8 ± 0.2
**Arterial hypertension, n (%)**	147 (82.1)
**Diabetes, n (%)**	71 (39.7)
**Smokers, n (%)**	50 (27.9)
**Chronic kidney disease, n (%)**	47 (26.3)
**Atrial fibrillation, n (%)**	48 (27.4)
**Coronary artery disease, n (%)**	62 (34.6)
**COPD, n (%)**	23 (12.9)
**EuroSCORE II**	4.5 (2.4–7.5)
**Total progression time, months**	43.0 (25.8–63.9)
**Progression: moderate → severe AS, months**	32.0 (18.0–47.6)
**Progression: severe AS → intervention, months**	3.7 (1.4–12.6)

Values are presented as frequency and percentage or mean and standard deviation/median and interquartile range. Abbreviations: AS, aortic stenosis; COPD, chronic obstructive pulmonary disease.

**Table 2:** Longitudinal changes in echocardiographic parameters across echocardiographic assessments (mixed-effects models).

N = 179	Moderate AS	Severe Asymptomatic AS	Severe Symptomatic AS	*p* Value
**Peak velocity, m/s**	3.3 (2.8–3.8)	4.1 (3.6–4.6)	4.2 (3.7–4.7)	**<0.001** **^1,2^**
**Max gradient, mmHg**	43.7 (41.3–46.1)	67.1 (63.8–70.4)	69.6 (67.2–71,9)	**<0.001** **^1^**^,2^
**Mean gradient, mmHg**	24.1 (22.7–25.5)	39 (37–40.9)	40.9 (39.4–42.3)	**<0.001** **^1,2^**
**AVA, cm^2^**	1.1 (1.1–1,2)	0.8 (0.8–0.9)	0.7 (0.7–0.8)	**<0.001** **^1,2,3^**
**Indexed AVA, cm^2^/m^2^**	0.6 (0.6–0.7)	0.5 (0.4–0.5)	0.4 (0.4–0.4)	**<0.001** **^1,2,3^**
**Stroke volume, mL**	77.2 (73.7–80.7)	75.3 (70.3–80.3)	70.3 (66.7–73.9)	**0.008** **^2^**
**Stroke volume index, mL/m^2^**	43.6 (41.7–45.5)	42.8 (40.1–45.6)	39.4 (37.5–41.4)	**0.034 ^2^**
**Transvalvular flow, mL/s**	251.9 (240.1–263.6)	239.2 (222.3–256)	233.1 (220.3–246)	0.076
**GLS, %**	−17.9 (−18.7–17.2)	−16.3 (−17.3–15.4)	−14.9 (−15.7–14.1)	**<0.001 ^2^** **^,3^**
**LVEF, %**	64.9 (63.4–66.4)	63.5 (61.4–65.5)	58.1 (56.6–59.6)	**<0.001** **^2,3^**
**LVEDV, mL**	83.5 (78–88.9)	83.6 (76.4–90.9)	100.4 (94.8–105.9)	**<0.001** **^2,3^**
**LVEDV index, mL/m^2^**	46 (43.2–48.8)	44.8 (41–48.6)	55.7 (52.9–58.5)	**<0.001** **^2,3^**
**LVESV, mL**	30.1 (26.9–33.3)	32.7 (28.7–36.8)	43.4 (40.2–46.7)	**<0.001** **^2,3^**
**LVESV index, mL/m^2^**	16.6 (15–18.3)	17.4 (15.3–19.5)	24 (22.3–25.7)	**<0.001** **^2,3^**
**LV mass, g**	202.5 (192–213.1)	214.8 (200.7–228.8)	217.3 (206.6–228)	0.091
**LV mass index, g/m^2^**	113.8 (108.4–119.2)	121.2 (113.8–128.7)	121.8 (116.3–127.3)	0.067
**LA volume, mL**	74.3 (67.7–80.9)	81.6 (73.5–89.8)	87.1 (80.4–93.7)	**<0.001** **^2^**
**LA volume index, mL/m^2^**	40.9 (37.3–44.5)	46.2 (42.7–50.8)	49.1 (45.4–52.8)	**<0.001** **^1,2^**
**sPAP, mmHg**	27.2 (25.1–29.2)	31.2 (28.3–34.1)	36.3 (34.1–38.4)	**<0.001** **^1,2,3^**
**TAPSE, cm**	2.3 (2.2–2.5)	2.1 (1.9–2.3)	2.1 (1.9–2.2)	**0.032** **^2^**
**E/e′ ratio**	13.3 (12.3–14.4)	14.3 (12.7–15.9)	14.2 (13.2–15.2)	0.310
**Significant MR, %**	15.2 (9.7– 20.6)	18.3 (12.6–24)	28 (19.8–36.8)	**0.018 ^2^**
**Significant TR, %**	12.3 (7.3–17.3)	14.9 (9.6–20.3)	17.6 (9.5–25.7)	0.418
**RVAc**	1.0 (0.9–1.2)	0.8 (0.7–0.9)	0.7 (0.5–0.8)	**0.002** **^1,2^**

Data are presented as estimated marginal means (95% confidence intervals) for continuous variables and marginal percentages (95% confidence intervals) for binary variables. Bold values indicate statistically significant pairwise differences after multiplicity adjustment. Abbreviations: AS, aortic stenosis; AVA, aortic valve area; GLS, global longitudinal strain; LA, left atrium; LV: left ventricle; LVEDV/LVESV, left ventricular end-diastolic/end-systolic volume; LVEF, left ventricular ejection fraction; MR, mitral regurgitation; sPAP, systolic pulmonary artery systolic pressure; TAPSE, tricuspid annular plane systolic excursion; TR, tricuspid regurgitation; RVAc, right ventricular–arterial coupling. ^1^ *p* < 0.05 after Bonferroni adjustment for comparison between moderate AS and severe asymptomatic AS. ^2^ *p* < 0.05 after Bonferroni adjustment for comparison between moderate AS and severe symptomatic AS. ^3^ *p* < 0.05 after Bonferroni adjustment for comparison between severe asymptomatic AS and severe symptomatic AS.

**Table 3:** Longitudinal changes in cardiac damage stages across echocardiographic assessments.

**Généreux staging system [10]**			
**Comparison**	**OR**	**95%** **CI**	***p*** **Value**
**Severe asymptomatic AS vs. Moderate AS**	1.7	1–2.9	0.057
**Severe symptomatic AS vs. Moderate AS**	5.2	3.2–8.6	<0.001
**Severe symptomatic AS vs. Severe asymptomatic AS**	3.1	1.8–5.4	<0.001
**Gutiérrez–Ortiz staging system [11]**			
**Comparison**	**OR**	**95%** **CI**	***p*** **Value**
**Severe asymptomatic AS vs. Moderate AS**	0.9	0.5–1.4	0.511
**Severe symptomatic AS vs. Moderate AS**	0.3	0.2–0.5	<0.001
**Severe symptomatic AS vs. Severe asymptomatic AS**	0.4	0.2–0.6	<0.001

Results are reported as odds ratios (ORs) with 95% confidence intervals (CIs), representing the odds of being in a higher damage stage. Abbreviations: AS, aortic stenosis; CI, Confidence Intervals; OR, odds ratio.

## Data Availability

Dataset available on request from the authors due to restrictions (patients’ privacy).

## Supplementary Materials

The following are available online at https://www.mdpi.com/article/10.3390/jcm15041575/s1; Table S1: STROBE Statement; Table S2: Recommendations to grade aortic stenosis severity; Table S3: Feasibility of key echocardiographic measures at each point; Table S4: Sensitivity analysis: repeated-measures ANOVA results for echocardiographic parameters across assessments; File S1: Stata v16 code for ordinal mixed models.

## Abbreviations

The following abbreviations are used in this manuscript:

AS	aortic stenosis
AVA	aortic valve area
CI	confidence interval
GLS	global longitudinal strain
IRB	Institutional Review Board
IQR	interquartile range
LAVI	left atrial volume index
LV	left ventricular;
LVOT	left ventricular outflow tract
LVEDVi/LVESVi	left ventricular end-diastolic/systolic volume index
LVEF	left ventricular ejection fraction
OR	odds ratio
RVAc	right ventricular–arterial coupling
sPAP	systolic pulmonary artery pressure
TTE	transthoracic echocardiography
TAPSE	tricuspid annular plane systolic excursion
TAVR	transcatheter aortic valve replacement
