# Supplementary material for: Evolution of Cardiac Damage Across Clinically Defined Stages of Aortic Stenosis in Patients Undergoing TAVR: A Single-Center Retrospective Cohort Study
Source: J Clin Med. 2026 Feb 17;15(4):1575. doi: 10.3390/jcm15041575 (PMC12941740; doi:10.3390/jcm15041575)
Supplement: Supplementary file 1 [file jcm-15-01575-s001.zip › Supplementary material/jcm-4091166-layout material (final 3).pdf]

Journal of
Clinical Medicine

MDPI

Supplementary material

Table S1. STROBE Statement (20)

	Item No	Recommendation
**Title and abstract**	1	(a) Indicate the study's design with a commonly used term in the title or the abstract: *page 1, lines 2-4 and page 1, lines 18-20*
		(*b*) Provide in the abstract an informative and balanced summary of what was done and what was found: *page 1, lines 15-35.*
**Introduction**		
Background/rationale	2	Explain the scientific background and rationale for the investigation being reported: *page 2, lines 40-76*
Objectives	3	State specific objectives, including any prespecified hypotheses: page 2 and 3, lines 78-82
**Methods**		
Study design	4	Present key elements of study design early in the paper: *page 3, lines 85-86*
Setting	5	Describe the setting, locations, and relevant dates, including periods of recruitment, exposure, follow-up, and data collection: *page 3, lines 86-94*
Participants	6	Give the eligibility criteria, and the sources and methods of selection of participants. Describe methods of follow-up: *page 3, lines 97-113*
Variables	7	Clearly define all outcomes, exposures, predictors, potential confounders, and effect modifiers. Give diagnostic criteria, if applicable: *pages 4 and 5, lines 120-187*
Data sources/ measurement	8	For each variable of interest, give sources of data and details of methods of assessment (measurement). Describe comparability of assessment methods if there is more than one group: *pages 4 and 5, lines 118-187*
Bias	9	Describe any efforts to address potential sources of bias: *pages 4 and 5, lines 118-181*
Study size	10	Explain how the study size was arrived at: *page 3, lines 98-99*
Quantitative variables	11	Explain how quantitative variables were handled in the analyses. If applicable, describe which groupings were chosen and why: *page 4, lines 152-181*
Statistical methods	12	(*a*) Describe all statistical methods, including those used to control for confounding: *pages 5 and 6, lines 188-223*
		(b) Explain how missing data were addressed: *page 5, lines 208-210*

		(c) Describe any sensitivity analyses: *page 5, lines 208-210*
**Results**		
Participants	13	(a) Report numbers of individuals at each stage of study—eg numbers potentially eligible, examined for eligibility, confirmed eligible, included in the study, completing follow-up, and analysed: *page 3, lines 111-114*
		(b) Give reasons for non-participation at each stage: *page 3, lines 110-113*
		(c) Consider use of a flow diagram: *pages 3 and 4, lines 115-117*
Descriptive data	14	(a) Give characteristics of study participants (eg demographic, clinical, social) and information on exposures and potential confounders: *page 6, lines 225-241*
		(b) Indicate number of participants with missing data for each variable of interest: *page 5, lines 179-181 and page 9, lines 314-318*
		(c) Summarise follow-up time (eg, average and total amount): *page 6, lines 232-241*
Outcome data	15	Report numbers of outcome events or summary measures over time: *page 7, lines 242-271; page 9, lines 300-313; and pages 9 and 10, lines 319-343*
Main results	16	Give unadjusted estimates and, if applicable, confounder-adjusted estimates and their precision (eg, 95% confidence interval). Make clear which confounders were adjusted for and why they were included: *pages 7 and 8, lines 274-287; and pages 10,11 and 12, lines 340-362.*
Other analyses	17	Report other analyses done—eg analyses of subgroups and interactions, and sensitivity analyses: *page 5, lines 208-210; and pages 8 and 9, lines 289-299*
**Discussion**		
Key results	18	Summarise key results with reference to study objectives: *page 12, lines 366-378*
Limitations	19	Discuss limitations of the study, taking into account sources of potential bias or imprecision. Discuss both direction and magnitude of any potential bias: *page 14: lines 450-473*
Interpretation	20	Give a cautious overall interpretation of results considering objectives, limitations, multiplicity of analyses, results from similar studies, and other relevant evidence: *pages 12, 13 and 14: lines 379-449*
Generalisability	21	Discuss the generalisability (external validity) of the study results: *page 13 and 14, lines 419-449*
**Other information**		
Funding	22	Give the source of funding and the role of the funders for the present study and, if applicable, for the original study on which the present article is based: *page 15, line 497*

Table S2. Recommendations to grade aortic stenosis severity (16)

	Aortic sclerosis	Mild	Moderate	Severe
**Maximum jet velocity (m/s)**	**≤ 2.5**	**2.6-2.9**	**3.0-4.0**	**≥ 4**
**Mean transvalvular gradient (mmHg)**	**-**	**< 20**	**20-40**	**≥ 40**
**Aortic valve area (AVA), cm^2**	**-**	**> 1.5**	**1.0-1.5**	**< 1.0**
**Indexed AVA (cm^2/m^2)**	**-**	**> 0.85**	**0.60-0.85**	**< 0.6**
**Dimensionless index**	**-**	**> 0.50**	**0.25-0.50**	**< 0.25**

LVOT diameter and PW-LVOT VTI were acquired at a consistent anatomic level across time points to minimize AVA staging artifacts related to LVOT measurement drift. Abbreviations: AVA, aortic valve area.

Table S3. Feasibility of key echocardiographic measures at each point

Measure	Moderate AS, n/N (%)	Severe asymptomatic AS, n/N (%)	Severe symptomatic AS, n/N (%)
**GLS available**	163/179 (91.1)	140/179 (78,2)	143/179 (79.9)
**TAPSE available**	136/179 (76)	156/179 (87.2)	167/179 (93.3)
**sPAP available**	167/179 (93.3)	140/179 (78,2)	144/179 (80.4)
**RVAc available**	131/179 (73.2)	128/179 (71.5)	130/179 (72.6)

Feasibility is reported as n/N (%), where N=179 represents the overall study cohort. For each time point, n denotes the number of patients with an available/quantifiable measurement. Measurements were considered missing if the parameter could not be quantified due to image quality/technical limitations. RVAc was defined as TAPSE/sPAP and could only be computed when sPAP whether TAPSE were estimable; therefore, RVAc was treated as missing when sPAP whether TAPSE were not measurable. Abbreviations: AS, aortic stenosis; GLS, global longitudinal strain; TAPSE, tricuspid annular plane systolic excursion; sPAP, systolic pulmonary artery systolic pressure; RVAc, right ventricular–arterial coupling.

Table S4. Sensitivity analysis: repeated-measures ANOVA results for echocardiographic parameters across assessments

N=179	Moderate AS	Severe asymptomatic AS	Severe symptomatic AS	P value
**Peak velocity, m/s**	3.3 ± 0.5	4.1 ± 0.5	4.2 ± 0.6	**<0.001**[1,2]
**Max gradient, mmHg**	43.7 ± 13.8	67.9 ± 14.5	69.5 ± 18.7	**<0.001**[1,2]
**Mean gradient, mmHg**	24.1 ± 8.0	39.6 ± 8.5	40.9 ± 11.4	**<0.001**[1,2]
**AVA, cm²**	1.1 ± 0.4	0.8 ± 0.3	0.8 ± 0.2	**<0.001**[1,2]
**Indexed AVA, cm²/m²**	0.6 ± 0.2	0.5 ± 0.2	0.4 ± 0.1	**<0.001**[1,2]
**Stroke volume, mL**	77.2 ± 25.3	74.8 ± 23.8	70.2 ± 22.8	**0.023**[2]
**Stroke volume index, mL/m²**	43.6 ± 14.1	42.8 ± 13.8	39.4 ± 22.7	**0.008**[2]
**Transvalvular flow, mL/s**	252.0 ± 77.2	238.2 ± 68.9	232.4 ± 87.7	0.078
**GLS, %**	-18.1 ± 4.8	-17.1 ± 4.1	-15.0 ± 4.7	**<0.001**[2,3]
**LVEF, %**	64.9 ± 10.1	64.6 ± 10.4	58.1 ± 10.2	**<0.001**[2,3]
**LVEDV, mL**	83.6 ± 35.6	81.4 ± 35.8	100.6 ± 38.9	**<0.001**[2,3]
**LVEDV index, mL/m²**	46.0 ± 20.8	46.0 ± 18.5	55.9 ± 18.3	**<0.001**[2,3]
**LVESV, mL**	30.2 ± 18.6	30.3 ± 20.6	43.6 ± 24.7	**<0.001**[2,3]
**LVESV index, mL/m²**	16.6 ± 10.2	17.1 ± 10.9	24.1 ± 12.2	**<0.001**[2,3]
**LV mass, g**	202.5 ± 76.8	213.8 ± 76.9	218.3 ± 63.8	0.114
**LV mass index, g/m²**	113.8 ± 40.8	121.7 ± 40.2	122.3 ± 30.6	0.070
**LA volume, mL**	74.4 ± 50.4	79.9 ± 37.1	87.4 ± 43.8	**0.029**[2]
**LA volume index, mL/m²**	40.9 ± 27.4	45.8 ± 27.4	49.4 ± 24.1	**0.006**[2]
**sPAP, mmHg**	27.2 ± 13.1	31.5 ± 15.4	37.1 ± 14.1	**<0.001**[2]
**TAPSE, cm**	2.3 ± 1.3	2.1 ± 0.4	2.1 ± 0.5	**0.036**[2]
**E/e′ ratio**	13.2 ± 5.6	13.7 ± 7.5	14.1 ± 5.3	0.531
**Significant MR, n [%]**	26 [14.9]	32 [18.1]	40 [22.3]	0.087
**Significant TR, n [%]**	21 [12.1]	27 [15.0]	29 [16.7]	0.578
**RVAc**	1.0 ± 0.9	0.8 ± 0.4	0.7 ± 0.3	**0.001**[1,2]

Data are presented as mean ± standard deviation or n (%). Bold values indicate statistically significant differences.

Abbreviations: AS, aortic stenosis; AVA, aortic valve area; GLS, global longitudinal strain; LA, left atrium; LVEDV/LVESV, left ventricular end-diastolic/end-systolic volume; LVEF, left ventricular ejection fraction; MR, mitral regurgitation; PASP, pulmonary artery systolic pressure; TAPSE, tricuspid annular plane systolic excursion; TR, tricuspid regurgitation; RVAc, right ventricular–arterial coupling.

[1] $p < 0.05$ after Bonferroni adjustment for comparison between moderate AS and severe asymptomatic AS.

[2] $p < 0.05$ after Bonferroni adjustment for comparison between moderate AS and severe symptomatic AS.

[3] $p < 0.05$ after Bonferroni adjustment for comparison between severe asymptomatic AS and severe symptomatic AS.

File S1. Stata v16 code for ordinal mixed models

```
* Cardiac damage staging across AS time points
****************************************************
version 16.0
set more off

* --- Data structure (expected):
* Long format: one row per patient (TAVI_N) per time point (ECO)
* ECO coded as: 0=Moderate AS, 1= Severe asymptomatic AS, 2=Symptomatic severe AS (pre-TAVR)
* stage_gutierrez coded 0–3 (4 categories)
* stage_genereux coded 0–4 (5 categories)

* Ensure ECO reference category is Moderate AS (ECO=0)
fvset base 0 ECO

****************************************************
* A) Gutiérrez staging system (0–3)
****************************************************
meologit stage_gutierrez i.ECO || TAVI_N:, nolog

* Odds ratios (OR) with 95% CI for each time point vs reference (ECO=0)
lincom 1.ECO, eform      // Severe asymptomatic AS vs Moderate AS
lincom 2.ECO, eform      // Severe symptomatic AS vs Moderate AS
lincom 2.ECO - 1.ECO, eform    // Severe symptomatic AS vs Severe asymptomatic AS

* Marginal predicted probabilities by time point (optional)
margins ECO

* Post hoc pairwise comparisons of marginal predictions (optional)
margins ECO, post
pwcompare ECO, mcompare(bonferroni) effects

****************************************************
* B) Généreux staging system (0–4)
****************************************************
meologit stage_genereux i.ECO || TAVI_N:, nolog

lincom 1.ECO, eform
lincom 2.ECO, eform
lincom 2.ECO - 1.ECO, eform

margins ECO
margins ECO, post
pwcompare ECO, mcompare(bonferroni) effects
****************************************************
```
