# Supplementary material for: Evolution of Cardiac Damage Across Clinically Defined Stages of Aortic Stenosis in Patients Undergoing TAVR: A Single-Center Retrospective Cohort Study
Source: J Clin Med. 2026 Feb 17;15(4):1575. doi: 10.3390/jcm15041575 (PMC12941740; doi:10.3390/jcm15041575)
Supplement: Supplementary file 1 [file jcm-15-01575-s001.zip › Supplementary material/jcm-4091166-layout material (final 3).docx]

Supplementary material

**Table S1.** STROBE Statement (20)

|  | **Item No** | **Recommendation** |
| --- | --- | --- |
| **Title and abstract** | 1 | (a) Indicate the study’s design with a commonly used term in the title or the abstract: *page 1, lines 2-4 and page 1, lines 18-20* |
|  |  | (*b*) Provide in the abstract an informative and balanced summary of what was done and what was found: *page 1, lines 15-35.* |
| **Introduction** | | |
| Background/rationale | 2 | Explain the scientific background and rationale for the investigation being reported: *page 2, lines 40-76* |
| Objectives | 3 | State specific objectives, including any prespecified hypotheses: page 2 and 3, lines 78-82 |
| **Methods** | | |
| Study design | 4 | Present key elements of study design early in the paper: *page 3, lines 85-86* |
| Setting | 5 | Describe the setting, locations, and relevant dates, including periods of recruitment, exposure, follow-up, and data collection: *page 3, lines 86-94* |
| Participants | 6 | Give the eligibility criteria, and the sources and methods of selection of participants. Describe methods of follow-up: *page 3, lines 97-113* |
| Variables | 7 | Clearly define all outcomes, exposures, predictors, potential confounders, and effect modifiers. Give diagnostic criteria, if applicable: *pages 4 and 5, lines 120-187* |
| Data sources/ measurement | 8 | For each variable of interest, give sources of data and details of methods of assessment (measurement). Describe comparability of assessment methods if there is more than one group: *pages 4 and 5, lines 118-187* |
| Bias | 9 | Describe any efforts to address potential sources of bias: *pages 4 and 5, lines 118-181* |
| Study size | 10 | Explain how the study size was arrived at: *page 3, lines 98-99* |
| Quantitative variables | 11 | Explain how quantitative variables were handled in the analyses. If applicable, describe which groupings were chosen and why: *page 4, lines 152-181* |
| Statistical methods | 12 | (*a*) Describe all statistical methods, including those used to control for confounding: *pages 5 and 6, lines 188-223* |
|  |  | (b) Explain how missing data were addressed: *page 5, lines 208-210* |
|  |  | (c) Describe any sensitivity analyses: *page 5, lines 208-210* |
| **Results** | | |
| Participants | 13 | (a) Report numbers of individuals at each stage of study—eg numbers potentially eligible, examined for eligibility, confirmed eligible, included in the study, completing follow-up, and analysed: *page 3, lines 111-114* |
|  |  | (b) Give reasons for non-participation at each stage: *page 3, lines 110-113* |
|  |  | (c) Consider use of a flow diagram: *pages 3 and 4, lines 115-117* |
| Descriptive data | 14 | (a) Give characteristics of study participants (eg demographic, clinical, social) and information on exposures and potential confounders: *page 6, lines 225-241* |
|  |  | (b) Indicate number of participants with missing data for each variable of interest: *page 5, lines 179-181 and page 9, lines 314-318* |
|  |  | (c) Summarise follow-up time (eg, average and total amount): *page 6, lines 232-241* |
| Outcome data | 15 | Report numbers of outcome events or summary measures over time: *page 7, lines 242-271; page 9, lines 300-313; and pages 9 and 10, lines 319-343* |
| Main results | 16 | Give unadjusted estimates and, if applicable, confounder-adjusted estimates and their precision (eg, 95% confidence interval). Make clear which confounders were adjusted for and why they were included: *pages 7 and 8, lines 274-287; and pages 10,11 and 12, lines 340-362.* |
| Other analyses | 17 | Report other analyses done—eg analyses of subgroups and interactions, and sensitivity analyses: *page 5, lines 208-210; and pages 8 and 9, lines 289-299* |
| **Discussion** | | |
| Key results | 18 | Summarise key results with reference to study objectives: *page 12, lines 366-378* |
| Limitations | 19 | Discuss limitations of the study, taking into account sources of potential bias or imprecision. Discuss both direction and magnitude of any potential bias: *page 14: lines 450-473* |
| Interpretation | 20 | Give a cautious overall interpretation of results considering objectives, limitations, multiplicity of analyses, results from similar studies, and other relevant evidence: *pages 12, 13 and 14: lines 379-449* |
| Generalisability | 21 | Discuss the generalisability (external validity) of the study results: *page 13 and 14, lines 419-449* |
| **Other information** | | |
| Funding | 22 | Give the source of funding and the role of the funders for the present study and, if applicable, for the original study on which the present article is based: *page 15, line 497* |

**Table S2.** Recommendations to grade aortic stenosis severity (16)

|  | Aortic sclerosis | Mild | Moderate | Severe |
| --- | --- | --- | --- | --- |
| Maximum jet velocity (m/s) | $\boldsymbol{\leq}$ **2.5** | **2.6-2.9** | **3.0-4.0** | $\boldsymbol{\geq}$ **4** |
| Mean transvalvular gradient (mmHg) | **-** | $\mathbf{<}$**20** | **20-40** | $\boldsymbol{\geq}$ **40** |
| Aortic valve area (AVA), cm² | **-** | $\mathbf{>}$ **1.5** | **1.0-1.5** | $\mathbf{<}$**1.0** |
| Indexed AVA (cm²/m²) | **-** | $\mathbf{>}$ **0.85** | **0.60-0.85** | $\mathbf{<}$**0.6** |
| Dimensionless index | **-** | $\mathbf{>}$ **0.50** | **0.25-0.50** | $\mathbf{<}$**0.25** |

LVOT diameter and PW-LVOT VTI were acquired at a consistent anatomic level across time points to minimize AVA staging artifacts related to LVOT measurement drift. Abbreviations: AVA, aortic valve area.

**Table S3.** Feasibility of key echocardiographic measures at each point

| Measure | Moderate AS, n/N (%) | Severe asymptomatic AS, n/N (%) | Severe symptomatic AS, n/N (%) |
| --- | --- | --- | --- |
| GLS available | 163/179 (91.1) | 140/179 (78,2) | 143/179 (79.9) |
| TAPSE available | 136/179 (76) | 156/179 (87.2) | 167/179 (93.3) |
| sPAP available | 167/179 (93.3) | 140/179 (78,2) | 144/179 (80.4) |
| RVAc available | 131/179 (73.2) | 128/179 (71.5) | 130/179 (72.6) |

Feasibility is reported as n/N (%), where N=179 represents the overall study cohort.  For each time point, n denotes the number of patients with an available/quantifiable measurement. Measurements were considered missing if the parameter could not be quantified due to image quality/technical limitations. RVAc was defined as TAPSE/sPAP and could only be computed when sPAP whether TAPSE were estimable; therefore, RVAc was treated as missing when sPAP whether TAPSE were not measurable. Abbreviations: AS, aortic stenosis; GLS, global longitudinal strain; TAPSE, tricuspid annular plane systolic excursion; sPAP, systolic pulmonary artery systolic pressure; RVAc, right ventricular–arterial coupling.

**Table S4.** Sensitivity analysis: repeated-measures ANOVA results for echocardiographic parameters across assessments

| N=179 | Moderate AS | Severe asymptomatic AS | Severe symptomatic AS | P value |
| --- | --- | --- | --- | --- |
| Peak velocity, m/s | 3.3 ± 0.5 | 4.1 ± 0.5 | 4.2 ± 0.6 | **<0.001^1,2^** |
| Max gradient, mmHg | 43.7 ± 13.8 | 67.9 ± 14.5 | 69.5 ± 18.7 | **<0.001^1^**^,2^ |
| Mean gradient, mmHg | 24.1 ± 8.0 | 39.6 ± 8.5 | 40.9 ± 11.4 | **<0.001^1,2^** |
| AVA, cm² | 1.1 ± 0.4 | 0.8 ± 0.3 | 0.8 ± 0.2 | **<0.001^1,2^** |
| Indexed AVA, cm²/m² | 0.6 ± 0.2 | 0.5 ± 0.2 | 0.4 ± 0.1 | **<0.001^1,2^** |
| Stroke volume, mL | 77.2 ± 25.3 | 74.8 ± 23.8 | 70.2 ± 22.8 | **0.023^2^** |
| Stroke volume index, mL/m² | 43.6 ± 14.1 | 42.8 ± 13.8 | 39.4 ± 22.7 | **0.008^2^** |
| Transvalvular flow, mL/s | 252.0 ± 77.2 | 238.2 ± 68.9 | 232.4 ± 87.7 | 0.078 |
| GLS, % | -18.1 ± 4.8 | -17.1 ± 4.1 | -15.0 ± 4.7 | **<0.001^2,3^** |
| LVEF, % | 64.9 ± 10.1 | 64.6 ± 10.4 | 58.1 ± 10.2 | **<0.001^2,3^** |
| LVEDV, mL | 83.6 ± 35.6 | 81.4 ± 35.8 | 100.6 ± 38.9 | **<0.001^2,3^** |
| LVEDV index, mL/m² | 46.0 ± 20.8 | 46.0 ± 18.5 | 55.9 ± 18.3 | **<0.001^2,3^** |
| LVESV, mL | 30.2 ± 18.6 | 30.3 ± 20.6 | 43.6 ± 24.7 | **<0.001^2,3^** |
| LVESV index, mL/m² | 16.6 ± 10.2 | 17.1 ± 10.9 | 24.1 ± 12.2 | **<0.001^2,3^** |
| LV mass, g | 202.5 ± 76.8 | 213.8 ± 76.9 | 218.3 ± 63.8 | 0.114 |
| LV mass index, g/m² | 113.8 ± 40.8 | 121.7 ± 40.2 | 122.3 ± 30.6 | 0.070 |
| LA volume, mL | 74.4 ± 50.4 | 79.9 ± 37.1 | 87.4 ± 43.8 | **0.029^2^** |
| LA volume index, mL/m² | 40.9 ± 27.4 | 45.8 ± 27.4 | 49.4 ± 24.1 | **0.006^2^** |
| sPAP, mmHg | 27.2 ± 13.1 | 31.5 ± 15.4 | 37.1 ± 14.1 | **<0.001^2^** |
| TAPSE, cm | 2.3 ± 1.3 | 2.1 ± 0.4 | 2.1 ± 0.5 | **0.036^2^** |
| E/e′ ratio | 13.2 ± 5.6 | 13.7 ± 7.5 | 14.1 ± 5.3 | 0.531 |
| Significant MR, n [%] | 26 [14.9] | 32 [18.1] | 40 [22.3] | 0.087 |
| Significant TR, n [%] | 21 [12.1] | 27 [15.0] | 29 [16.7] | 0.578 |
| RVAc | 1.0 ± 0.9 | 0.8 ± 0.4 | 0.7 ± 0.3 | **0.001^1,2^** |

Data are presented as mean ± standard deviation or n (%). Bold values indicate statistically significant differences.

**Abbreviations:** AS, aortic stenosis; AVA, aortic valve area; GLS, global longitudinal strain; LA, left atrium; LVEDV/LVESV, left ventricular end-diastolic/end-systolic volume; LVEF, left ventricular ejection fraction; MR, mitral regurgitation; PASP, pulmonary artery systolic pressure; TAPSE, tricuspid annular plane systolic excursion; TR, tricuspid regurgitation; RVAc, right ventricular–arterial coupling.

¹ *p* < 0.05 after Bonferroni adjustment for comparison between moderate AS and severe asymptomatic AS.
² *p* < 0.05 after Bonferroni adjustment for comparison between moderate AS and severe symptomatic AS.
³ *p* < 0.05 after Bonferroni adjustment for comparison between severe asymptomatic AS and severe symptomatic AS.

**File S1.** Stata v16 code for ordinal mixed models

* Cardiac damage staging across AS time points

*******************************************************

version 16.0

set more off

* --- Data structure (expected):

* Long format: one row per patient (TAVI_N) per time point (ECO)

* ECO coded as: 0=Moderate AS, 1= Severe asymptomatic AS, 2=Symptomatic severe AS (pre-TAVR)

* stage_gutierrez coded 0–3 (4 categories)

* stage_genereux coded 0–4 (5 categories)

* Ensure ECO reference category is Moderate AS (ECO=0)

fvset base 0 ECO

*******************************************************

* A) Gutiérrez staging system (0–3)

*******************************************************

meologit stage_gutierrez i.ECO || TAVI_N:, nolog

* Odds ratios (OR) with 95% CI for each time point vs reference (ECO=0)

lincom 1.ECO, eform      // Severe asymptomatic AS vs Moderate AS

lincom 2.ECO, eform      // Severe symptomatic AS vs Moderate AS

lincom 2.ECO - 1.ECO, eform   // Severe symptomatic AS vs Severe asymptomatic AS

* Marginal predicted probabilities by time point (optional)

margins ECO

* Post hoc pairwise comparisons of marginal predictions (optional)

margins ECO, post

pwcompare ECO, mcompare(bonferroni) effects

*******************************************************

* B) Généreux staging system (0–4)

*******************************************************

meologit stage_genereux i.ECO || TAVI_N:, nolog

lincom 1.ECO, eform

lincom 2.ECO, eform

lincom 2.ECO - 1.ECO, eform

margins ECO

margins ECO, post

pwcompare ECO, mcompare(bonferroni) effects

*******************************************************
