# Supplementary material for: Evolution of Cardiac Damage Across Clinically Defined Stages of Aortic Stenosis in Patients Undergoing TAVR: A Single-Center Retrospective Cohort Study
Source: J Clin Med. 2026 Feb 17;15(4):1575. doi: 10.3390/jcm15041575 (PMC12941740; doi:10.3390/jcm15041575)
Supplement: Supplementary file 1 [file jcm-15-01575-s001.zip › Supplementary material/back matter (final).docx]

**Supplementary Materials:** The following are available online at https://www.mdpi.com/article/doi/s1; Table S1: STROBE Statement; Table S2: Recommendations to grade aortic stenosis severity; Table S3: Feasibility of key echocardiographic measures at each point; Table S4: Sensitivity analysis: repeated-measures ANOVA results for echocardiographic parameters across assessments; File S1: Stata v16 code for ordinal mixed models.

**Author Contributions:** Conceptualization, F.I.; methodology, M.R.-R. and C.O.; software, M.R.-R.; validation, M.R.-R., C.O. and F.I.; formal analysis, M.R.-R. and C.O., investigation, M.R.-R., C.O., F.I., S.G.-A., P.J.-Q., E.P.-O., L.N.-F., and J.A.d.A.; resources, J.A.d.A, M.R.-R. and C.O.; data curation, M.R.-R., F.I.; writing—original draft preparation, M.R.-R.; writing—review and editing, C.O.; visualization, M.R.-R. P.J.-Q., E.P.-O., L.N.-F.; supervision, C.O.; project administration, M.R.-R. and C.O. All authors have read and agreed to the published version of the manuscript.

**Funding:** This research received no external funding.

**Institutional Review Board Statement:** Our study was conducted in accordance with the Declaration of Helsinki and was approved by the Ethics Committee of our institution (code 20/238-E [5-02-2020]). All patients provided written informed consent for the procedures.

**Informed Consent Statement:** Informed consent was obtained from all subjects involved in the study.

**Data Availability Statement:** Dataset available on request from the authors due to restrictions (patients’ privacy).

**Acknowledgments:** During the preparation of this manuscript, no scientific content was generated or altered by the AI. The authors have reviewed and edited the output and take full responsibility for the content of this publication.

**Conflicts of Interest:** The authors declare no conflicts of interest.
